# Implementation Strategies and Outcomes for Whole-System Violence Reduction: A Case Study from Northern Ireland

**DOI:** 10.3390/bs16050684

**Published:** 2026-04-30

**Authors:** Claire Hazelden, Christopher Farrington

**Affiliations:** 1 Strategic Investment Board, The Kelvin, Belfast BT1 6DE, Northern Ireland, UK; 2 Executive Programme on Paramilitarism and Organised Crime, Stormont Estate, Belfast BT4 3TA, Northern Ireland, UK; christopher.farrington@justice-ni.gov.uk

**Keywords:** violence, implementation, architecture, governance, trauma, collaboration, prevention, exploitation, adoption, sustainability

## Abstract

Background: Governments increasingly seek whole-system, public-health approaches to prevent serious youth violence. However, there is limited empirical evidence on how such approaches are implemented and sustained in complex, post-conflict settings characterised by coercive control, political instability, and fragmented system ownership. Aim: This study examines the Executive Programme on Paramilitarism and Organised Crime (EPPOC) in Northern Ireland as a system-level implementation architecture for addressing serious youth violence, with a focus on how coordinated action was enabled, constrained, and adapted over time. Methods: We conducted an embedded qualitative case study of EPPOC using systematic analysis of programme documentation, independent evaluations, oversight reports, and population-level data spanning nine years of delivery. Implementation science frameworks (ERIC, Proctor’s implementation outcomes, and CFIR) were applied retrospectively as analytic lenses to examine implementation strategies, outcomes, and contextual determinants. Results: EPPOC demonstrated strong implementation outcomes in acceptability and adoption across statutory and community sectors, supported by cross-government governance, trauma-informed workforce development, and shared learning systems. Penetration and sustainability were more variable and constrained by political instability, short-term funding cycles, uneven departmental ownership, and coercive community conditions. Conclusions: The findings suggest that the most transferable element of EPPOC is not individual interventions but the implementation architecture that enabled coordinated, trauma-responsive action across government in a highly complex environment. This architecture represents a potentially replicable design pattern for jurisdictions seeking to address serious youth violence where traditional programme models struggle to operate.

## 1. Introduction

Serious youth violence associated with organised criminal activity represents one of the most persistent and complex social harms facing contemporary public policy. In many jurisdictions, young people’s involvement in violence is shaped by cumulative exposure to trauma, socio-economic deprivation, educational exclusion, and peer and community dynamics rather than by individual risk factors alone. As a result, there is growing recognition that isolated interventions or enforcement-led responses are insufficient to address the structural and relational conditions that sustain harm, particularly for those already exposed to the highest levels of risk.

In Northern Ireland, these challenges are compounded by the enduring legacy of conflict and the continued presence of paramilitary and organised criminal groups. For some children and young people, pathways into serious violence and exploitation are entangled with coercive control, community intimidation, and crime that constrain help-seeking and undermine trust in statutory services. These dynamics create distinctive implementation challenges for prevention and intervention efforts, including fragmented ownership, limited legitimacy, and heightened risk for those delivering and accessing support.

The Executive Programme on Paramilitarism and Organised Crime (EPPOC) was established to address these conditions through a coordinated, cross-government response spanning prevention, safeguarding, criminal justice, and community-based activity. EPPOC seeks to align policy, funding, governance, and delivery across multiple departments and sectors to reduce paramilitary harm, including serious youth violence and child criminal exploitation. Its design reflects a whole-system ambition that prioritises coordination, shared accountability, and long-term capacity-building.

Despite increasing policy emphasis on whole-system approaches to serious youth violence, the existing literature leaves a critical gap in understanding how such approaches are implemented and sustained in practice, particularly in post-conflict settings characterised by political instability, coercive community dynamics, and low institutional trust. Most research focuses either on the effectiveness of discrete interventions or on descriptive accounts of violence and victimisation, offering limited insight into the system-level implementation architectures that enable coordinated action across government, statutory services, and communities over time. There is especially little empirical work applying implementation science frameworks to analyse cross-government violence-reduction systems operating under conditions of ongoing trauma and contested authority.

This paper addresses this gap by presenting a qualitative case study of the Executive Programme on Paramilitarism and Organised Crime (EPPOC) as a system-level implementation architecture in Northern Ireland. Using implementation science as an analytic lens, rather than an ex ante design template, the study contributes practice-based evidence on how whole-system approaches to serious youth violence are authorised, coordinated, adapted, and constrained in a complex post-conflict environment. In doing so, it shifts attention from individual interventions to the implementational conditions that make sustained, cross-system violence reduction possible, and offers a potentially transferable design pattern for jurisdictions facing similarly high-harm and coercive contexts.

The contribution of this study is therefore not an evaluation of intervention effectiveness, but an analysis of how a whole-system violence-reduction architecture is implemented and sustained in a highly constrained real-world setting.

### 1.1. Literature Review

Serious youth violence is widely conceptualised as a complex public health problem, shaped by interacting individual, relational, community, and structural determinants rather than by isolated risk factors alone (World Health Organization, 2014, 2024). Exposure to violence, adverse childhood experiences, school exclusion, substance misuse, poverty, and local conditions interact over time, producing pathways into higher-harm violence that are resistant to single-agency solutions (Walsh & Redmond, 2026). As a result, there is growing consensus that prevention and response require coordinated, multi-sector systems spanning health, education, justice, social care, and community services.

Although prevention research has advanced for universal and secondary-tier provision, the evidence base remains uneven at the tertiary end of harm. Large-scale evidence syntheses consistently show stronger evidence for parenting programmes, school-based approaches, and skills-focused interventions than for activity targeting young people already embedded in serious violence, exploitation, or organised criminality (Open Innovation Team, 2023; Youth Endowment Fund, 2024). This gap is particularly pronounced where violence is reinforced by coercion, fear, and organised criminal structures, as these conditions can negatively impact engagement, credibility, and sustainability. These limitations have increased policy interest in whole-system or ecosystem approaches that prioritise shared problem framing, aligned outcomes, and sustained enabling infrastructure rather than isolated projects (Open Innovation Team, 2023). Despite the literature endorsing these approaches in principle, there remains limited guidance describing how such systems are implemented and sustained in practice, especially in settings characterised by political instability and/or contested authority.

International literature reinforces the characterisation of serious youth violence as a complex social and public health problem requiring multicomponent intervention strategies. In Canada and Australia, for example, violence affecting Indigenous children and young people is widely framed as the product of structural inequality, intergenerational trauma, and systemic marginalisation, with evidence consistently emphasising the limited effectiveness of enforcement-led responses in the absence of culturally grounded, community-governed systems of support (Perreault, 2022; Moore et al., 2022; Trevethan & Maxwell, 2023).

Across these contexts, effective responses are described in the literature as those that integrate prevention, healing, relational support, and ongoing multi-agency coordination. Yet much of this literature is descriptive or evaluative at programme level, and offers only limited insights into the system-level implementation mechanisms that enable coordination, adaptation, and sustainability (particularly where violence is reinforced by fear and potential threat).

Serious youth violence in Northern Ireland is impacted by both the legacy of the conflict and the ongoing existence of violent paramilitary groups. Research consistently demonstrates that exposure to conflict-related and post-agreement violence has generated high levels of intergenerational and community-level trauma, shaping trust, informal control, and norms around violence in some areas (McAlister et al., 2013; Walsh & Cunningham, 2023). These dynamics constrain help-seeking, undermine statutory legitimacy in some areas, and place voluntary and community organisations under direct or indirect threat (Northern Ireland Affairs Committee, 2024; Sturgeon et al., 2024). As a result, harms are likely to be under-reported, and system responses to these issues are not always comprehensive.

Research, including a population-level study, has consistently demonstrated that paramilitary influence, intimidation, and the normalisation of threat remain salient features of the social environment in which many children and young people grow up (McAlister et al., 2013; Walsh et al., 2025a).

This literature highlights the nature and consequences of serious youth violence in Northern Ireland but offers comparatively little analysis of how trauma-informed, preventative, and multisystem responses are operationalised in practice.

Implementation science provides a set of frameworks for understanding how evidence-informed interventions and models are adopted, enacted, and sustained (E. Proctor et al., 2011; Curran, 2020). Although much of the literature has focused on clinical and organisational innovations, there is increasing recognition of its relevance to complex public sector and social interventions, including violence prevention and public health systems (Curran, 2020). The key frameworks we are using are the Expert Recommendations for Implementing Change (ERIC), Proctor et al.’s taxonomy of implementation outcomes, and the Consolidated Framework for Implementation Research (CFIR).

The Expert Recommendations for Implementing Change (ERIC) offer a taxonomy of 73 implementation strategies spanning planning, financing, restructuring, education, facilitation, and evaluative learning (Powell et al., 2015; Powell et al., 2017).

Proctor and colleagues’ taxonomy of implementation outcomes distinguishes implementation success (e.g., acceptability, adoption, feasibility, fidelity, penetration, and sustainability) from service or population outcomes (E. Proctor et al., 2011). This distinction is particularly important in serious youth violence contexts, where attribution is methodologically challenging.

The Consolidated Framework for Implementation Research (CFIR) complements these approaches by organising determinants of implementation across five domains: intervention characteristics, outer setting, inner setting, characteristics of individuals, and implementation process (Damschroder et al., 2009; Damschroder et al., 2022). CFIR is particularly well-suited to analysing system-level initiatives operating in volatile or constrained environments, as it explicitly incorporates political context, organisational capacity, workforce capability, and external shocks. These factors are highly relevant to the Northern Ireland setting, where historical legacies, community pressures, and political instability actively shape how interventions are perceived, adopted, and sustained.

Notably, much of the implementation science literature assumes relatively stable governance conditions and relatively linear pathways from evidence to impact. There remains limited work applying ERIC, Proctor, or CFIR to cross-government programmes operating under prolonged political instability, constrained institutional legitimacy, and coercive conditions. In such contexts, adaptation is inevitable rather than optional; however, implementation science cautions that unmanaged adaptation risks diluting intervention intent and obscuring causal mechanisms. Emerging work in violence prevention suggests that implementation processes themselves, such as relational continuity, trusted interagency coordination, and sustained facilitation, can shape engagement and risk trajectories, particularly in settings where fear, under disclosure, or coercive control constrain access to statutory services (Barranco et al., 2022; Walsh, 2021). Nonetheless, there is a paucity of detailed case studies that examine how these mechanisms are generated and maintained across whole systems over time.

This literature highlights a clear gap between the widespread endorsement of whole-system approaches to serious youth violence and the limited empirical understanding of how such systems are implemented and sustained in complex, post-conflict environments. While there is strong conceptual agreement on the need for coordinated, trauma-responsive systems, there is insufficient analysis of the implementation architectures that enable cross-government action under conditions of coercion, political volatility, and institutional constraint.

This study addresses this gap by examining EPPOC as a system-level implementation architecture, using implementation science frameworks as an analytic lens to explore how strategies, outcomes, and contextual determinants interact in practice. In doing so, it shifts attention from individual interventions to the implementational conditions that make sustained, whole-system violence reduction possible in the most challenging settings.

### 1.2. What Is EPPOC?

The Executive Programme on Paramilitarism and Organised Crime (EPPOC) was established in 2016 as a cross-government initiative designed to reduce harms associated with paramilitary activity, coercive control, organised criminality, and serious youth violence (NI Executive, 2016). Its remit spans prevention, safeguarding, criminal justice, and community-based interventions, with a particular emphasis on serious youth violence and child criminal exploitation. EPPOC operates through a portfolio of coordinated interventions delivered by multiple government departments, statutory agencies, and voluntary and community organisations. EPPOC emerged from a political agreement and initially supported projects identified by an Independent Panel (Alderdice et al., 2016). Its early architecture reflected pragmatic governance and programme-management choices rather than an explicit implementation or public-health framework. As a result, EPPOC has evolved iteratively in response to operational pressures, political volatility, and emerging evidence, rather than following a linear design pathway.

Over time, EPPOC has come to function as a ‘whole system’ response, applying public health principles, meaning it treats serious youth violence as a preventable ‘disease’ with risk and protective factors, and emphasises prevention, data-driven strategies, and multi-agency collaboration. This is akin to the approach used in the World Health Organization’s Violence Prevention Alliance, focusing on early intervention and addressing root causes (World Health Organization, 2022).

This ambition has been accompanied by persistent challenges with delivery, accountability and measurement. The central claim of this article is that EPPOC’s “intervention” is not only the funded portfolio of services; it is the conceptual and operational architecture that makes cross-government delivery possible. While this has supported alignment and collaboration, it has also been vulnerable to political instability, short-term funding cycles and staff turnover.

EPPOC is therefore examined as a case of system-level implementation in a constrained post-conflict environment. This framing allows critical examination of how implementation strategies, outcomes and context interact over time, and of the limits as well as the possibilities of whole system violence reduction approaches in such environments.

### 1.3. Aim and Questions

Our aim is to contribute a practice-based account of how whole-system approaches to serious youth violence can be implemented, adapted, and sustained in a complex post-conflict setting. The analysis applies implementation science frameworks to understand system-level processes, outcomes and constraints.

Rather than evaluating the effectiveness of individual interventions, we have focused on EPPOC’s role as implementation architecture and the mechanisms that enable or limit coherent action.

We seek to do this by answering three interrelated questions: how was EPPOC implemented as a system-level intervention?; what implementation strategies were used?; and what factors influenced implementation outcomes?

## 2. Materials and Methods

### 2.1. Design

The implementation of EPPOC as a contemporary whole-system response to paramilitary harm, organised criminality, and serious youth violence in Northern Ireland is the case study. The case study design is appropriate where the aim is to understand a real-world phenomenon in depth, particularly when the boundaries between the phenomenon and its context are not clearly separable, and where multiple sources of evidence are needed to support explanation and interpretation (Yin, 2018, p. 50).

EPPOC was selected because it represents an unusual example of cross-government, prevention-oriented system change operating in a complex setting with a high level of trauma.

Our purpose is explanatory and practice-oriented: to understand how EPPOC operated as an implementation architecture over time, rather than to evaluate its component parts.

### 2.2. Case Study

The case in this study is EPPOC, conceptualised as a bounded programme-level system intervention. The programme-level architecture was the primary unit of analysis, which comprises its governance and authorising environment, enabling framework, project portfolio, portfolio management, shared outcomes/benefits approach, collaborative infrastructure, and learning/evaluation system.

To examine variation in how the system operated in practice, we also examined embedded units, corresponding to delivery components within the EPPOC portfolio. These included funded projects and thematic workstreams (spanning prevention, early intervention, crisis response, recovery, and enforcement). Analysing these embedded units enabled comparison across different parts of the delivery system while retaining EPPOC’s programme architecture as the central “case” of interest. This was to avoid the case study describing a set of project case studies as if they were disconnected.

The case was bounded temporally across Phase One (2016–2021) and Phase Two (2021–present), with documentary evidence examined for the entire period. Contextually, Northern Ireland’s post-conflict environment, including inter and intra-community dynamics, the effects of trauma, including conflict-related trauma, and political instability, was treated as integral to the case, because these conditions shaped both implementation and the extent to which implementation outcomes could be achieved.

### 2.3. Data Sources

The study draws primarily on systematic analysis of documentary evidence produced between 2016 and 2023. Documents were treated as data rather than as administrative records, and were analysed to reconstruct how EPPOC’s implementation architecture developed, adapted, and functioned over time. The documents originate from a range of sources, of which the majority are both publicly available and independently produced. Data sources were synthesised into four analytical categories:

Foundational and strategic documents, including policy agreements, programme initiation materials, and action plans, which were used to understand EPPOC’s original remit, assumptions, and governance design (Alderdice et al., 2016; NI Executive, 2016; Northern Ireland Office, 2015).

Programme level implementation and governance materials, such as internal reports, programme implementation, delivery updates, discussion papers and progress towards outcomes (e.g., EPPOC, 2020, 2021).

Independent evaluations and thematic studies, including process and project evaluations, action research, outcome evaluations, and thematic studies on cross-cutting issues relevant to EPPOC (e.g., EPPOC & PHA, 2025; Walsh, 2022b, 2023c; Walsh et al., 2025c). External reviews were used to assess implementation processes, perceived effects, and areas of constraint across different parts of the system (e.g., Independent Reporting Commission, 2018, 2019, 2020, 2021, 2022, 2023, 2024, 2025; Northern Ireland Affairs Committee, 2024).

Population Level and administrative Data, including survey findings and official statistics related to paramilitary harm and youth violence, which were used to contextualise programme activity and triangulate implementation claims (e.g., NISRA, 2025a, 2025b).

Documentary analysis was conducted systematically, with documents appraised for relevance, evidentiary value, and intended purpose. Analysis focused on identifying patterns in implementation strategies, governance arrangements, adaptation over time, and reported outcomes, rather than on compiling a comprehensive inventory of programme outputs. Documentary evidence was read alongside the authors’ practice-based knowledge derived from direct involvement in programme governance and delivery. This insight was used to interpret operational context and link evidence across time, while analytic interpretation was disciplined by implementation science frameworks and triangulated against independent evaluations and external oversight reports to mitigate proximity bias.

### 2.4. Analytic Approach: Implementation Mapping

Using these evidence sources, we developed an analytic “map” of EPPOC that links Programme architecture, implementation strategies, implementation outcomes, and contextual determinants in a coherent narrative. Implementation science frameworks were used as a structured analytic lens to organise and interpret evidence, rather than as an a priori design guide for EPPOC. The analysis was conducted iteratively and focused on understanding how the programme functioned in practice as a system-level implementation architecture.

In this paper, ‘implementation architecture’ is defined as the enabling mechanisms that make a system-level model adoptable, deliverable, and sustainable. Operationally, it comprises the linked mechanisms for authorisation and governance, resourcing and funding flows, coordination and support infrastructure, and learning and adaptation loops that embed practice across multiple settings. This is distinct from MSP/PRINCE2, which provides methods for planning and controlling delivery; implementation architecture refers to the wider system mechanisms that enable uptake, consistent enactment, and institutionalisation beyond the life of a managed programme.

The analysis proceeds in three steps, using complementary implementation science frameworks.

Step 1: Implementation strategies. Documentary evidence was examined to identify EPPOC’s main programme-level activities and enabling functions. These were extracted and mapped to the Expert Recommendations for Implementing Change (ERIC) compilation of implementation strategies, using the nine-category structure described in the ERIC concept-mapping work to organise and describe the Programme’s strategy “bundles” (Powell et al., 2015). The intent was not to claim that all strategies were implemented formally, but to provide a structured vocabulary to describe how EPPOC used levers such as funding, mandate, infrastructure, facilitation, training, evaluative learning, and adaptation to enable cross-system implementation.

Step 2: Implementation outcomes (E. Proctor et al., 2011). EPPOC’s implementation performance was assessed using Proctor and colleagues’ taxonomy of eight implementation outcomes. This enabled a clear separation between implementation success (e.g., acceptability in contested contexts; penetration into practice; sustainability constraints) and broader population outcomes that are influenced by multiple strategic dependencies.

Step 3: Determinants and constraints. To explain variation in implementation performance and the translation of implementation gains into wider change, we used the Consolidated Framework for Implementation Research (CFIR) to organise determinants across domains (Damschroder et al., 2009, 2022). Particular emphasis was placed on outer-setting volatility (including periods of political instability and macro shocks), coercive community conditions, and partial cross-system ownership as determinants shaping penetration and sustainability.

Together, this approach provides the organising logic for the Findings section, which presents EPPOC as an implementation architecture in practice and traces how strategies and mechanisms relate to observed implementation outcomes under real-world constraints.

### 2.5. Ethics, Confidentiality, and Safe Reporting

This article is written to protect individuals and operational sensitivities. The analysis focuses on Programme-level architecture and mechanisms rather than identifiable case material. Examples are presented at the level of implementation functions and system determinants, avoiding disclosure of sensitive operational details. Ethical review was not required for this secondary analysis of Programme documentation and anonymised evaluative materials; however, all reporting was undertaken in line with organisational information governance requirements.

### 2.6. Authors’ Roles in EPPOC

The authors both hold professional roles within the EPPOC Programme Team and were involved in aspects of design, governance and delivery. This positionality provides access to detailed documentation, longitudinal Programme insights and operational knowledge that would be difficult to obtain externally. At the same time, such proximity introduces limitations that shape what can be observed, interpreted and claimed.

First, the analysis is necessarily shaped by the evidentiary record produced by EPPOC and by its external, independent reviewers. While these sources are extensive and include both independent evaluations and oversight reports, they reflect institutional perspectives and priorities. As a result, the analysis is better suited to examining how EPPOC functioned as an implementation architecture than capturing a full range of lived experience.

Second, proximity to operations can normalise features that might otherwise be subject to deeper external critique. Although implementation science frameworks (ERIC, Proctor, and CFIR) were used to discipline interpretation and to surface variation, they cannot fully eliminate the interpretative effects of insider status.

Third, the authors’ roles within EPPOC limit the extent to which the study can adopt an adversarial analytic stance. The analysis cannot claim neutrality, but does offer a theoretically informed, practice-based interpretation of how system-level implementation operated under real-world constraints. Claims are therefore bounded: the study illuminates implementation processes, tensions, and trade-offs, but cannot establish causal attribution or fully independent judgement of programme intent or impact.

To mitigate these limitations, interpretation was structured using established implementation science frameworks and triangulated with independent evaluations and data: whenever possible, we cite findings from independent evaluation reports or oversight bodies (e.g., the Independent Reporting Commission’s annual reports) to support or challenge our interpretations. Where interpretation goes beyond documented evidence, this is presented as reflective assessment. Nonetheless, the analysis remains situated and partial, reflecting both the strengths and the constraints of insider research within complex public-sector systems.

## 3. Findings (Results): EPPOC as Implementation Architecture in Practice

This Results section reports findings from documentary analysis structured around three questions:

- how was EPPOC implemented as a system-level intervention?
- what implementation strategies were used?
- what factors influenced implementation outcomes?

Throughout, we distinguish evidence of implementation strength from evidence of limitation or partial achievement, and we highlight variation where this was visible in the data.

### 3.1. EPPOC as a System-Level Intervention

The EPPOC is a cross-government initiative established in Northern Ireland in 2016 to reduce the harms associated with paramilitary activity, coercive control, organised criminality, and serious youth violence. EPPOC functioned at the lead organisation, but the key partners that delivered interventions were Northern Ireland government departments, policing and justice agencies, statutory agencies, local councils, community and voluntary sector organisations, and academic partners.

EPPOC is best understood as a cross-government system-change programme delivered through multi-agency collaboration and operating across the full spectrum of prevention and protection. It provides a rare, real-world example of how government can attempt to coordinate trauma-responsive, evidence-informed, whole-system action in a context shaped by conflict legacy, structural trauma, and organised exploitation.

Over its lifespan, the Programme evolved from a traditional action-plan model into a system-level intervention rooted in public health principles. Phase One (2016–2021) focused on establishing core delivery structures and demonstrating cross-agency coordination at speed (EPPOC, 2020). Phase Two (2021–present) deliberately shifted to prevention, system coherence, and outcomes alignment across government.

EPPOC now functions as a whole-system intervention spanning prevention, early intervention, crisis response, and recovery, with dedicated activity targeting youth exploitation and serious harm. This makes EPPOC an instructive case for analysing implementation in complex youth-violence environments.

### 3.2. The Implementation Architecture

In EPPOC, systems thinking supplements the public health model by providing a bespoke toolbox for dealing with complexity. While public health offers a blueprint, systems thinking introduces the adaptive, partnership-driven mechanisms needed to make that blueprint workable in contested, post-conflict environments.

EPPOC’s implementation architecture comprises five interlocking components that structure coordinated action: cross-government governance; a shared outcomes and benefits framework; collaborative infrastructure; learning systems; and a trauma-informed approach.

#### 3.2.1. Cross-Government Governance

Central to EPPOC is a governance structure that intentionally spans departments, statutory agencies and community/voluntary partners (Northern Ireland Affairs Committee, 2024). Comparative research (Ansell & Gash, 2008; Emerson et al., 2012) indicates that this is a relatively rare type of governance model. The EPPOC model has:

- brought together authority, funding, and statutory levers across multiple Northern Ireland departments and agencies under a shared cross-Executive mandate, supported by formal governance and external scrutiny arrangements rather than single-department ownership (EPPOC, 2022; Independent Reporting Commission, 2025);
- addressed fragmented responses to paramilitary harm, youth violence, and coercive control by embedding multi-agency delivery and shared problem-solving across statutory and community sectors, rather than relying on isolated departmental or enforcement responses (EPPOC, 2022; Northern Ireland Affairs Committee, 2024); and
- established sustained cross-agency decision-making structures at both strategic and local levels, including cross-Executive governance arrangements and place-based partnership mechanisms (EPPOC, 2022).

However, data indicate these governance arrangements have been resource-intensive and relationally demanding. Building trust and sharing ownership across departments and agencies took time and often depended on consistent interpersonal engagement rather than formal mandate. In some instances, EPPOC funded dedicated capacity within departments to accelerate relationship building, coordination and shared problem solving. This was effective in addressing initial barriers, but it does demonstrate the costs involved in developing cross-governmental work, as well as raising questions about sustainability.

The collaborative environment was reinforced through Programme-wide public awareness campaigns designed to challenge the normalisation of paramilitary harm and support shared understanding among the public and frontline services (see www.endingtheharm.com (accessed on 25 February 2026)). EPPOC placed explicit emphasis on data-informed decision-making, supported by action research, monitoring, and evaluation structures that feed learning back into programme design and delivery (EPPOC, 2022; Hazelden, 2025).

#### 3.2.2. A Codesigned, Shared Outcomes and Benefits Framework

A Benefits Realisation Framework anchors EPPOC as a system intervention, aligning project-level work with measurable outcomes, codesigned and agreed by all involved. The Framework:

- identifies the changes required at project level to help to drive population-level impact (across keeping people safe, community resilience and individual protective factors);
- connects diverse projects and multiple methodologies to a shared theory of change; and
- helps to structure governance, planning and adaptation.

This framework operationalises implementation-science guidance by distinguishing implementation outcomes from population outcomes and enabling system-level coherence (E. Proctor et al., 2011).

**Figure 1:**
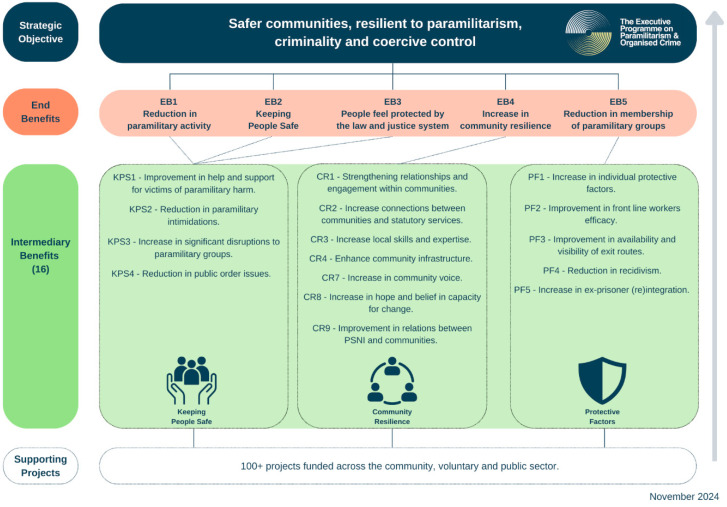
The EPPOC Benefits Realisation Framework.

The benefits framework (Figure 1) is used by cross-departmental groups to align activity, delivery partners to connect project-level work to system-level aims, and analysts to test assumptions and triangulate evidence. Political and senior leaders rely on it to understand system direction and constraints.

By making outcomes explicit, measurable and testable, the framework enables EPPOC to function as a coherent, whole-of-government system intervention rather than a set of siloed initiatives. It translates the public-health model into operational practice. In a contested, post-conflict environment, this outcomes-focused backbone is essential for sustaining coordinated action and understanding what it takes to generate and maintain system-level change.

#### 3.2.3. Collaborative Infrastructure

EPPOC’s collaborative infrastructure ensures consistent multi-agency responses on youth vulnerability, coercive control, and community tensions. This includes:

- cross-agency forums for problem-solving and sharing ‘what works’, particularly around barriers that no agency could resolve alone;
- local planning mechanisms;
- shared tools and language around trauma, coercion and protective factors.

These support a common understanding of risk and enable practitioners across sectors to make sense of complex harm in similar ways. This has been particularly important in this setting where legitimacy, trust, and perceptions of statutory services vary widely across communities (Northern Ireland Affairs Committee, 2024).

#### 3.2.4. Learning Systems

EPPOC’s collaborative structures are the engine of its adaptive learning system, which distributes evidence across the system. Information flowed both upward (into governance and strategy) and outward (across delivery partners), which helps maintain coherence and shared understanding in an environment that is complex and adaptive. Over time, EPPOC developed both a substantial evidence base and a culture of learning. This includes:

- systematic monitoring of projects, relevant issues, the policy environment and wider strategic enablers;
- action research and project-level evaluations over multiple years (Coyle et al., 2022; Walsh, 2022a, 2022c, 2023a, 2023b, 2024a, 2024b; Walsh & Cunningham, 2023);
- structured annual reviews of benefits across the Programme, resulting in an overall contribution analysis of the Programme’s impact; and
- thematic studies on drug-related intimidation (EPPOC & PHA, 2025); child criminal exploitation (Walsh, 2022b; Walsh et al., 2025c), and Adverse Childhood Experiences (Walsh et al., 2025a, 2025b).

This learning system enabled EPPOC to identify and respond to emerging patterns, including those related to youth violence (Northern Ireland Affairs Committee, 2024). It also facilitated adaptive iteration as research and evidence enable a better understanding of both the issues and the efficacy of interventions.

The learning system represented one of the Programme’s most significant implementation assets; however, it was resource-intensive and required sustained analytical capability, long-term relationships and consistent engagement from delivery partners. Effectiveness was therefore tied to relational factors, including trust and openness. In several instances, procurement processes slowed commissioning, resulting in time lags between emerging issues and formal learning outputs. This acted as a constraint in terms of responsiveness.

These findings suggest EPPOC’s learning system was a deliberately constructed and intentionally maintained capability, reliant on investment, building and maintaining relationships, and sustained effort.

#### 3.2.5. A Trauma-Informed Approach

A trauma-informed approach emerged as one of EPPOC’s core pillars. Rather than treating trauma as an individual attribute, EPPOC understands it as a structural determinant (i.e., a set of social, economic, and institutional conditions that pattern exposure to harm and shape access to safety and support) that influences behaviour, service engagement, community trust, and institutional decision-making. A trauma-informed lens requires recognising the pervasive impact of trauma across individuals, families, communities and systems, and creating environments that are safe, predictable, and empowering for those affected. This is critical for tertiary-end youth violence, where coercion, exploitation, and community fear routinely shape help-seeking and service engagement.

Drawing on recognised frameworks (Substance Abuse and Mental Health Services Administration, 2014), partners embedded trauma-responsive practices through:

- delivery of accessible training across sectors;
- introducing organisational development support and practice standards; and
- strengthening frontline workers’ capability to respond to coercion, exploitation and adversity in safe, consistent and sustainable ways.

These mechanisms are associated with successful implementation in complex systems and have provided a common foundation for more compassionate and effective responses to harm across the Programme (Mooney et al., 2024).

#### 3.2.6. Constraints and Weaknesses

While EPPOC’s implementation architecture enabled coordination across multiple departments and sectors, the evidence also highlights structural constraints that limited what it could deliver in practice. These shaped pace, reach, and long-term durability.

A recurring limitation concerned access to system-wide data and unresolved data-sharing protocols. These constraints reduced the Programme Team’s ability to develop a fully data-driven public-health approach and limited opportunities to triangulate programme-level learning with wider administrative datasets (for example, education, health, or justice system data). Although partial workarounds were developed through evaluation, thematic research, and contribution analysis, the absence of an integrated data infrastructure constrained real-time system learning and reduced visibility of cumulative and cross-sector effects.

Attempts to strengthen information flows from communities and to develop shared data or intelligence mechanisms were heavily contingent on Programme Team capacity. Where analytical or coordination capacity was stretched, progress slowed or stalled. This illustrates that EPPOC’s learning and coordination functions relied on both formal structures and on sustained analytical and relational labour that was not evenly distributed or fully embedded across the wider system.

More fundamentally, the architecture frequently operated in a context where Programme capacity substituted for core system capability. Dedicated EPPOC resources convened partners, brokered information, sustained coordination, and managed risk in ways that could not be readily absorbed by departments facing competing priorities, workforce pressures, and fiscal constraints. While this enabled delivery in the short to medium term, it also exposed fragility in embedding system-level functions where ownership and resourcing remained external to departmental baselines.

Finally, funding and governance conditions constrained long-term planning and mainstreaming. Repeated short-term funding cycles and reliance on ring-fenced investment limited the extent to which successful practices could be mainstreamed as business-as-usual. As a result, the implementation architecture proved highly effective as a stabilising and enabling mechanism, but less effective as a vehicle for fully transferring responsibility into the wider system.

### 3.3. Implementation Strategies and Mechanisms

This section applies an implementation-science lens to describe how EPPOC has been implemented in practice. It shows how, in this case, Programme management structures can operate like implementation strategies.

An implementation strategy is a theory-informed set of ‘methods or techniques designed to increase the adoption, implementation, and sustainability’ of interventions in real-world settings (E. K. Proctor et al., 2013, p. 2). ERIC (Expert Recommendations for Implementing Change) is a comprehensive list of 73 discrete implementation strategies identified in implementation science research and offers a consensus-based compilation that spans planning, education, financing, restructuring, quality management, and policy levers (Powell et al., 2015, 2017). This taxonomy clarifies terminology and strengthens conceptual coherence so practitioners and systems can more deliberately select, tailor, and combine strategies to improve implementation outcomes. Waltz et al. (2015) mapped these 73 strategies into nine categories.

We have taken these categories to describe the EPPOC implementation strategy. The full list is in Appendix A; however, we have identified three dominant ‘bundles’ of ERIC strategies that were significant for EPPOC’s implementation, which are presented here in order of importance: authorise, fund, and stabilise; building the delivery system; and a learnable and adaptable system. ERIC is used comparatively to distinguish strategies that appeared to generate the strongest implementation gains from strategies that were weaker, incomplete, or constrained.

#### 3.3.1. Authorise, Fund and Stabilise

EPPOC’s primary lever was ‘utilising financial strategies’ by providing targeted investment to enable cross-agency delivery. The Programme is jointly funded by the Northern Ireland Executive and the United Kingdom Government, and its annual budget is currently around £16m. This joint commitment and significant investment created implementation momentum in the initial phases and subsequently enabled continuity of delivery.

However, it is worth noting that repeated short-term funding cycles linked to governmental budgetary cycles have acted as a structural constraint on longer-term planning.

#### 3.3.2. Building the Delivery System

A second dominant set of strategies focused on ‘building stakeholder interrelationships’, building ‘change infrastructure’, ‘providing interactive assistance’, and ‘training and educating stakeholders’ (described here as ‘building the delivery system’).

Strengthening stakeholder interrelationships has been pivotal to the Programme’s strategy, second only to the initial financial investment. The Fresh Start Agreement catalysed a coalition of motivated early adopters. In Phase One (2016–2021), the Programme Team prioritised building consensus on key concepts (such as lawfulness and ‘transition’ from paramilitarism) by convening and facilitating stakeholder discussions and producing position papers.

Phase Two (2021–date) shifted to more focused engagement with delivery partners, beginning with the codesign of the shared benefits framework. This has supported reflection, shared learning, and joint problem-solving. High-profile public awareness campaigns and research events have promoted knowledge sharing beyond immediate stakeholders, drawing interest from the media and members of the public. Recognising that it would be unsustainable to rely on a ‘coalition of the willing’, leadership was expanded within key Departments, ensuring structured coordination and expertise-driven leadership across the Programme.

The Programme Team assembled the infrastructure required for system-wide change and secured ministerial and departmental mandates that established EPPOC as a cross-government priority (The Executive Office, 2025). It also established and maintained administrative structures to create mandates for change.

As described in the Implementation Architecture section, EPPOC prioritised workforce development by delivering trauma-informed training and stakeholder education across statutory, voluntary, and community sectors (Mooney et al., 2024). This was complemented by a comprehensive suite of educational resources, including toolkits, e-learning modules, and guidance documents, distributed widely to support adaptation in diverse settings.

The aim of these strategies was to build shared understanding, consistent practice, and system-level alignment.

#### 3.3.3. Learnable and Adaptable System

EPPOC used ‘evaluative and iterative strategies’ to support adaptation, and ‘adapted and tailored to context’ using a variety of strategies while attempting to improve data visibility and shared understanding of harms and interventions.

A comprehensive approach to evaluation was developed in response to the original Panel Report’s recommendation, evolving from individual project assessments with varying standards to a coordinated Action Research model. EPPOC aimed to support practitioners in three key areas of implementation: evaluating process delivery; conducting qualitative research with users at project and thematic levels; and aligning outcome measurement with the Programme’s overarching framework. There were also efforts to build capacity with smaller, local organisations to carry out research and evaluation for EPPOC.

While the majority of EPPOC’s portfolio has remained consistent, there has been investment in new approaches and projects to test innovations and understand potential for scalability. Some of these approaches are based on evidence from elsewhere, such as hospital navigators or the combined law enforcement and public health approach to drug-related intimidation in Ireland (DRIVE, 2021), while others are new approaches based on evidence of emerging need, such as gender-based projects and child criminal exploitation approaches (Walsh, 2022b, 2024b). Feedback was actively sought from service users and those in the community, and this contributed to evaluations and portfolio learning. Iterative “test-and-learn” cycles also helped to refine project approaches.

Flexibility has been key: EPPOC adjusted methods based on factors like levels of coercion, local capacity, rural-urban differences, gendered experiences, and changing threats. EPPOC’s design has also been informed by risk factor research, i.e., studies identifying factors that increase the likelihood of youth offending (such as school exclusion, substance misuse, and adverse childhood experiences) as well as by trauma theory (Public Health England, 2019). This has informed context-specific interventions and initiatives, such as agile funds, to ensure the Programme responds quickly to new or emerging circumstances.

The authors, as part of the Programme Team, used data and evidence to support projects and scan for emerging issues. The aim of these practices was to maintain coherence while accommodating diversity across the system.

### 3.4. Implementation Outcomes

Implementation outcomes describe how well a model, intervention, or system is taken up, enacted, and embedded in real-world settings. E. Proctor et al.’s (2011) taxonomy provides a structured lens for analysing EPPOC’s performance as a whole-system approach to reducing paramilitary harm, coercive control, and serious youth violence. EPPOC’s structures were explicitly intended to improve implementation outcomes across sectors through a system-wide architecture. This section evaluates evidence of EPPOC’s implementation performance using Proctor et al.’s eight outcomes. Evidence from nine years of independent evaluations, contribution analysis, independent thematic studies, and partner feedback indicates that EPPOC achieved substantial implementation gains, with distinctive strengths in acceptability and adoption given the contested and coercive context. Throughout this section, we are referring to the implementation outcomes from the EPPOC system-level model rather than from a single intervention.

#### 3.4.1. Acceptability

Proctor et al. define acceptability as ‘the perception among implementation stakeholders that a given treatment, service, practice, or innovation is agreeable, palatable, or satisfactory’ (E. Proctor et al., 2011, p. 67). EPPOC has faced significant acceptability challenges related to the sensitivities of addressing paramilitary harm. Until the Programme’s establishment, paramilitary activity was not widely discussed or addressed outside of security or law enforcement circles, and the harms which paramilitaries caused to individuals and communities were not systemically understood or prioritised (EPPOC, 2020, p. 57). Paramilitaries created fear and intimidation and perpetrated violence, which, understandably, made service providers (both in the community and statutory sectors) reticent about being seen overtly to tackle the issues. In addition, delivery agents and service providers were reluctant to be perceived as delivering a programme perceived as addressing a political issue in a divided society. EPPOC has consistently framed paramilitarism and associated violence in terms of harm, trauma, exploitation, and prevention rather than identities or group affiliation, reflecting a public-health and trauma-informed orientation (EPPOC, 2022; Hazelden, 2025). This framing is reinforced through the Programme’s public awareness campaigns, and the widespread use of shared concepts such as vulnerability, protective factors, and coercive control across materials (see, for example, www.endingtheharm.com; Hazelden, 2025).The conscious effort to focus on the harms caused by paramilitaries rather than their role in the Troubles or their political identities matched with the lived experience of many, particularly as paramilitaries have increasingly been seen as organised criminals responsible for crimes including the supply and distribution of drugs (Sturgeon et al., 2024, p. 2). EPPOC’s public awareness campaign supported this by challenging the normalisation of paramilitary activity and harm (EPPOC, 2024). This had direct consequences for addressing issues connected to youth violence. For example, ‘youth recruitment’ into paramilitary organisations had been normalised, but a conscious effort to reframe this as Child Criminal Exploitation (NICCY, 2021) challenged mental models and improved the acceptability of interventions. For example, in 2024, the Departments of Health, Justice and Education launched an Action Plan on Child Criminal Exploitation that explicitly referenced the work of EPPOC (DoH, 2024).

Emphasising a trauma-informed, prevention-oriented approach has aligned with the ethos of relevant sectors (EPPOC, 2022). EPPOC frontline practitioners have reported increased confidence in responding to trauma, coercive control, and youth exploitation; teachers and youth workers endorsed the relevance of training; and community organisations strongly supported the move away from solely enforcement-based approaches (Walsh et al., 2025c; Mooney et al., 2024).

The Programme also made important alterations to its framing to address acceptability. For example, the original Panel Report included a recommendation on ‘lawfulness’ as an underpinning concept, but feedback from communities and young people said this was contested, seen as patronising, and indicated that they were a problem (EPPOC, 2020, p. 59). In Phase Two, the focus on lawfulness was removed, and instead a strengths-based approach seeking to build personal and community resilience was taken (Hazelden, 2025). Maybe most importantly, the Programme’s name was changed from the ‘Tackling paramilitary activity, criminality, and organised crime Programme’ to the ‘Executive Programme on Paramilitarism and Organised Crime’ because many were uncomfortable with being asked to ‘tackle’ paramilitaries.

These shifts functioned as implementation actions to increase fit and legitimacy. While there is still some understandable reluctance to badge projects as ‘tackling paramilitarism’, the authors have noted that delivery partners in general are now more confident with their role in the overall effort, and see the Programme as providing important strategic and policy cover for their conviction to address the harms caused by these groups.

#### 3.4.2. Adoption

Proctor et al. define adoption as: ‘the intention, initial decision, or action to try or employ an innovation or evidence-based practice. Adoption also may be referred to as ‘‘uptake.’’’ (E. Proctor et al., 2011, p. 69). Organisational adoption, in the context of EPPOC, refers to the extent to which statutory and partner organisations have incorporated the Programme’s public-health framing, shared priorities, and coordination mechanisms into their routine decision-making, planning, and inter-agency working, rather than treating EPPOC as an external or time-limited funding programme. Evidence of organisational adoption was observed across policing, education, health, youth services, and community and voluntary sector organisations, reflected in participation in EPPOC-linked governance structures, shared fora, and joint problem-solving processes. Adoption did not take the form of uniform changes to frontline delivery, but was primarily expressed through the uptake of shared trauma-informed, harm-focused, and prevention-oriented framings, alongside engagement in cross-agency approaches to interpreting and responding to risk.

For some organisations, EPPOC was described as providing an authorising framework that legitimised, connected, and amplified work they were already attempting to progress, particularly in relation to early intervention and support for vulnerable young people. In this sense, the Programme enabled existing practice to be more visible and systemically supported, rather than replacing it.

This does not necessarily indicate wholesale transformation, but rather a shift in professional orientation and system positioning from isolated or marginal activity towards more coordinated, prevention-focused engagement across agencies. Such shifts are consistent with implementation research emphasising the importance of relationships between implementation actors and their context and of the importance of shared understanding and relational alignment (May et al., 2018; Best et al., 2016).

The mechanisms that EPPOC used to ensure and improve adoption included: developing a mandate, designing and implementing a clear programme structure, and providing support through training and evidence generation.

As with most complex system-level interventions, adoption was uneven across settings and evolved over time. In parts of the statutory system, competing priorities, constrained budgets, leadership turnover, and workforce shortages reduced capacity to engage fully with EPPOC structures.

#### 3.4.3. Appropriateness

Proctor et al. define appropriateness as ‘the perceived fit, relevance, or compatibility of the innovation or evidence-based practice for a given practice setting, provider, or consumer; and/or perceived fit of the innovation to address a particular issue or problem’ (E. Proctor et al., 2011, p. 69). Emerging from a political agreement, EPPOC’s genesis is highly relevant to its appropriateness. On one hand, the original panel report set out recommendations it felt were appropriate to the context, but at the time, there was a paucity of evidence on how to deal with post-conflict violence generally and paramilitarism specifically. The primary mechanism that EPPOC used to improve appropriateness was its research and evaluative learning to better match interventions to underlying needs within a public-health approach.

The current model has attempted to use its research and data agenda to create a much closer fit between needs and interventions. Northern Ireland’s high levels of trauma exposure, intergenerational adversity, paramilitary coercion, and socio-economic inequality match closely with EPPOC’s emphasis on trauma-informed practice, whole of government partnership delivery, and preventative public health approaches. Stakeholders repeatedly identify EPPOC as uniquely suited to the complex, cross-system nature of paramilitary harm and youth exploitation (Walsh et al., 2025c). Community-based partners have also noted the Programme’s alignment with lived experience and its capacity to navigate localised dynamics of intimidation and fear (Walsh et al., 2025c). The independent reviews by the IRC noted that the programme’s focus on areas with the highest paramilitary activity was appropriate, targeting resources to communities under coercion.

#### 3.4.4. Costs

Proctor et al. define cost as both the resources required to implement an intervention and the financial consequences that follow (E. Proctor et al., 2011, p. 69). EPPOC’s investment of circa £16m per annum is the primary mechanism of change in this outcome area. EPPOC’s investment is consistent with that expected from change management interventions: relatively high upfront investment, expectations of long-term cost avoidance and savings, and a value-added system infrastructure to embed changes.

This investment is the mechanism by which delivery partners provide services to fill critical gaps, preventing escalation into high-cost crisis responses, and supporting individuals who were unlikely to otherwise access statutory provision (e.g., NISRA, 2019). There is evidence that EPPOC has reduced pressure on some statutory services. Available evaluation and administrative data indicate service-system benefits consistent with reduced pressure across several parts of the public sector. The CONNECT hospital-based youth work intervention supported 1416 vulnerable young people across two Emergency Departments in a single year, of whom a majority (54%) were repeat attenders, illustrating both concentrated need and cumulative demand on emergency care services; the evaluation identifies reduction in hospital recidivism (repeat ED attendance) as the primary intended outcome of the intervention, alongside mechanisms such as reducing “walkaways” that place additional strain on both health services and police resources (Walsh, 2024a). Housing Executive administrative data show a sustained decline in recorded homelessness presentations arising from paramilitary intimidation, including a reduction in paramilitary-related intimidation cases from 377 in 2018/19 to 124 in 2022/23, indicating reduced pressure on homelessness and housing support services (Northern Ireland Housing Executive, 2024).

There is also evidence of reduced severity of public-order incidents in areas with EPPOC-funded diversion (Independent Reporting Commission, 2024). The Programme’s learning system has also supported better targeting of services and reduced duplication. However, despite consistent efforts to embed successful interventions, there is a significant risk for long-term sustainability, as without continued investment, many services will contract or disappear, and the positive financial consequences will not be consolidated in the long run.

#### 3.4.5. Feasibility

E. Proctor et al. (2011, p. 60) define feasibility as ‘the extent to which a new treatment, or an innovation, can be successfully used or carried out within a given agency or setting’. For EPPOC, feasibility relates to whether the Programme’s system-level model could be operationalised in the complexity of Northern Ireland’s political, economic, and social environment. Partners have consistently reported that the Programme’s design was workable in practice: it provided clear expectations, functional partnership mechanisms, and the flexibility to respond to emerging needs. The primary mechanisms for obtaining feasibility were: Programme structures to improve shared understanding; investment in frontline capability to improve practitioner self-efficacy; and adaptable delivery to local conditions and external shocks.

The Programme’s structures supported feasible and coherent delivery by providing clear governance, shared outcomes, and stable coordination mechanisms that partners could rely on even when wider conditions were unstable. These structures reduced ambiguity, clarified roles, and created predictable spaces for joint planning and problem solving. The Programme provided a workable model for managing complex harm and made it possible for diverse organisations to maintain consistent, connected delivery.

Wider feasibility was enhanced by EPPOC’s investment in frontline capability. The Programme supported extensive training, organisational-development work, mentoring, and specialist interventions. These equipped practitioners with the skills, confidence, and tools needed to deliver complex work in communities affected by multiple harms, with individuals experiencing complex needs. Numerous project evaluations reported that EPPOC-funded models were practicable to implement, appropriately resourced, and responsive to local need, particularly in supporting victims, young people at risk, and communities with low baseline infrastructure (for example, Coyle et al., 2022; Sturgeon & Bryan, 2020; Walsh, 2023a).

The Programme’s adaptive capacity made implementation feasible during periods of external uncertainty and shocks, such as the collapse of the Northern Ireland Executive, the COVID-19 pandemic, and major economic pressures. EPPOC partners, coordinated by the Programme Team, consistently adjusted approaches, timelines, and coordination structures, which helped maintain delivery by supporting practitioner self-efficacy, reducing ambiguity, strengthening collective confidence, and allowing teams to reshuffle effort. Adaptation increased stakeholders’ perception that the model was implementable and sustained the conditions that enabled action in turbulent environments, particularly for work involving young people exposed to coercive control and exploitation. These conditions show that adaptive capacity was not an optional enhancement, but the mechanism that enabled the Programme to remain feasible within a complex and often volatile system.

#### 3.4.6. Fidelity

Fidelity is defined as ‘the degree to which an intervention was implemented as it was prescribed in the original protocol or as it was intended by the program developers’ (E. Proctor et al., 2011, p. 69). EPPOC achieved strong fidelity in several core domains.

A trauma-informed approach was implemented broadly and consistently across sectors, with evaluations and practitioner testimony indicating clear patterns of practice change (Mooney et al., 2024). This is particularly important for supporting young people affected by coercive control or exploitation.

Fidelity to EPPOC’s collaborative and prevention-focused model can be understood in terms of consistent adherence to core principles and operating assumptions, rather than uniformity of delivery. Public documentation and external scrutiny indicate that, despite periods of political instability, the Programme maintained its cross-Executive governance structures, multi-agency orientation, and public-health framing, enabling core collaborative mechanisms to continue to function (Independent Reporting Commission, 2025).

Across Programme materials, research outputs, and public communications, there is sustained emphasis on prevention, early intervention, trauma, and protective factors, suggesting continuity in the Programme’s underlying theory of change even as delivery contexts evolved (Hazelden, 2025). Fidelity was not uniform across all settings. Community resilience projects showed greater variation, and areas affected by coercive control, weaker infrastructure, or limited statutory presence found adherence more challenging. Capacity pressures and staff turnover also reduced consistency because sustained fidelity requires stable teams, supervision, and organisational time. Some projects sought to meet higher levels of need than their intended tier within the public health framework, reflecting local realities in a resource-constrained system, but creating difficulties for maintaining fidelity across the full model.

Fidelity across EPPOC was shaped by reinforcing mechanisms that supported consistent practice at the system and project level. Clear governance structures and shared outcomes created predictability about roles, expectations, and standards, which encouraged partners to align their work with the intended model. Trauma-informed workforce development strengthened practitioner capability and confidence, increased the likelihood that frontline staff would apply core principles consistently, particularly in work involving young people exposed to coercive control or exploitation. Collaborative forums and routine meetings reinforced shared mental models, enabling practitioners to make decisions using the same frameworks and early intervention logic. The Programme’s learning infrastructure provided continuous cues for reflection and adjustment, helping partners maintain adherence over time. These mechanisms collectively supported fidelity by creating clarity, capability, shared understanding, and ongoing reinforcement, all of which made it more likely that the system would deliver in line with EPPOC’s design even in variable and resource-constrained contexts.

#### 3.4.7. Penetration

Penetration is defined as ‘the integration of a practice within a service setting and its subsystems’ (E. Proctor et al., 2011, p. 70). For EPPOC, penetration can be understood as the degree to which its approaches (encompassing projects, policies and practice) have become embedded. EPPOC’s approaches appear to have penetrated widely into practice, language and inter-agency working across statutory and community sectors, including improved connection pathways between communities and services. These gained significant traction across youth services, probation, schools, and community groups, supported by shared tools, action research, and cross-agency learning structures (Walsh, 2021). Penetration was also evident in the expansion of connections between communities and statutory services. Area-based partnership models facilitated engagement on sensitive issues, including the exploitation of young people by paramilitary groups, across sectors.

EPPOC’s penetration was driven by mechanisms that created a coherent architecture linking actors who had previously worked in isolation. Cross-government governance structures brought departments, policing bodies, youth services, and community organisations into regular joint decision-making, which normalised collaborative practice and embedded consistent responses to youth vulnerability. These structures enabled coordinated approaches to child criminal exploitation, public order issues, and victim support, ensuring that practitioners across sectors used compatible frameworks. The Programme’s support to the Paramilitary Crime Task Force played a similar role in specialised settings by enabling joint operations, strengthening cross-border cooperation, and encouraging widespread adoption of trauma-sensitive approaches within policing partners.

Penetration was also enabled by mechanisms that made EPPOC’s practices usable and credible at the local level. Routine multi-agency coordination, shared problem-solving, and consistent communication created predictability and built trust between statutory and community partners, which made agencies more willing to align with the Programme’s approach. Training, shared tools, and action research helped embed common language and practice, allowing youth-facing services, probation, schools, and community groups to integrate trauma-responsive and prevention-led methods into everyday work.

Although penetration varied across sectors and localities, the presence of EPPOC-linked structures and shared ways of working across multiple parts of the system enabled a broad group of organisations to adopt and sustain the Programme’s core principles. EPPOC’s collaborative and prevention-focused orientation became incorporated into routine inter-agency working among participating organisations.

Through this process, EPPOC contributed to greater coherence in how youth violence and coercive control were understood and addressed across agencies, supporting more aligned and less fragmented responses without implying uniform practice or consistent outcomes across all settings.

#### 3.4.8. Sustainability

Sustainability is defined as ‘the extent to which a newly implemented treatment is maintained or institutionalised within a service setting’s ongoing, stable operations’ (E. Proctor et al., 2011, p. 70). For EPPOC, sustainability concerns the extent to which practices, structures, and outcomes continue beyond the life of the Programme. In this case, long-term sustainability depends on the extent to which the wider system will take ownership and mainstream interventions which are working well.

EPPOC has achieved meaningful progress in embedding sustainable ways of working, and the associated cultural and behavioural shifts are likely to persist. However, EPPOC’s longer-term sustainability is limited by structural constraints. The Programme’s funded services remain dependent on its ring-fenced resources rather than being incorporated into departmental baselines, which leaves core activity vulnerable to annual funding pressures. Long-term embedding is also likely to be constrained by uneven adoption (discussed above), workforce capacity pressures, and the continued influence of coercive control. As a result, while EPPOC has established durable practice norms and collaborative habits, the permanence of these gains will depend on decisions and conditions outside the Programme’s direct control.

EPPOC promoted sustainability by creating the core conditions that allow practices and relationships to endure beyond Programme funding. The Programme embedded a shared language, consistent conceptual frameworks, and redesigned cross-agency relationships that made collaborative working more routine and predictable. EPPOC has also generated substantial evidence base that clarified what works in communities affected by paramilitarism and serious youth violence, giving organisations the insight and justification needed to maintain effective approaches over time. Together, these mechanisms built cultural and operational foundations that support longer-term sustainability, even in the absence of guaranteed resourcing.

#### 3.4.9. Synthesis

Across all implementation outcomes, the evidence points to a pattern of uneven but explicable system performance, rather than uniformly strong implementation. EPPOC’s implementation architecture enabled periods of coherent, coordinated practice, but many were contingent on specific conditions and/or were not consistently sustained across sectors or time.

Where implementation was strongest, it was supported by a combination of clear governance structures, shared outcomes, predictable coordination mechanisms and sustained investment in trauma-informed workforce development. Collaborative forums, local problem-solving structures, and regular multi-agency communication reinforced shared mental models and enabled partners to respond collectively to youth vulnerability and coercive control. A strong learning infrastructure, including evaluation, action research, and feedback loops, provided continuous cues for refinement and adaptation. Finally, the Programme’s adaptive capacity, especially during external shocks and political instability, supported feasibility, protected fidelity, and enabled penetration in varied delivery contexts. These mechanisms operated together to embed EPPOC’s model in everyday practice and contributed to the durability of cultural and behavioural change across the system.

However, these system-wide mechanisms did not translate into uniform implementation strength across the full delivery landscape. Consistent with the adoption and penetration findings above, engagement and depth of embedding varied across sectors and localities, shaped by workforce capacity constraints, competing statutory priorities, and leadership turnover. In practice, this meant that the same overarching EPPOC architecture could support strong enactment in some settings while remaining more intermittent or fragile in others. Relatedly, where EPPOC-funded activity remained alongside, rather than within, core departmental activity, implementation gains were more exposed to short-term and operational pressures, limiting the extent to which EPPOC’s aims and principles were fully institutionalised. These findings indicate that EPPOC’s implementation success depended on the quality of its architecture, and also on wider system readiness to absorb and maintain change. The evidence shows that a strong implementation architecture alone is likely to be insufficient to overcome weak systemic ownership or political volatility.

## 4. Discussion: What Is Replicable About EPPOC?

Governments increasingly seek whole-system approaches to prevent serious youth violence, yet there is limited evidence on how models can be implemented and sustained in complex, post-conflict environments. Without comparable case studies, it is challenging to make firm conclusions about the replicability of EPPOC; however, there are important observations on what has shaped and constrained success. The Consolidated Framework for Implementation Research (CFIR) (Damschroder et al., 2009, 2022) offers a useful way of highlighting key conditions, although a full CFIR analysis is beyond the scope of this article. Therefore, the discussion below draws on the evidence presented in this article and the CFIR domains to highlight factors likely to condition replicability.

### 4.1. Characteristics of Intervention

The characteristics of EPPOC, as a system-level intervention, are inherently complex. Replication requires the capability to manage a multi-component portfolio spanning prevention, early intervention, crisis response, victim support, community development, communications, and enforcement. Jurisdictions must assess whether they have the infrastructure for portfolio management, adaptive delivery, and continuous learning. EPPOC’s adaptability was one of its strengths, but this requires political authorisation, a stable delivery team with specialist skills, and tolerance for iterative change. The evidence base produced by EPPOC, including theory-based contribution analysis and multi-year evaluation, has strengthened implementation. Replicability is likely to be easier where systems value evidence and are both willing and able to generate and translate it into consistent cross-agency practice.

### 4.2. Outer-Setting

The outer setting, including the economic, political and social context (Damschroder et al., 2009), has shaped EPPOC more profoundly than any specific intervention decision (Northern Ireland Affairs Committee, 2024). Political instability, prolonged periods without an Executive, pressures on public finances, and macro shocks such as COVID-19 created sustained turbulence that influenced both implementation pace and the translation of implementation gains into population-level outcomes. A notable challenge was maintaining momentum given political instability; the suspension of the Northern Ireland Assembly and Executive at multiple points during the Programme’s life cycle slowed decision-making and created uncertainty for EPPOC. This shows how a fragile outer setting can undermine implementation.

In addition, the coercive influence of paramilitary and organised criminal groups creates contextual conditions that differ markedly from those underpinning many mainstream youth-violence models. Research on the lived experiences of young people in post-conflict Northern Ireland highlights how fear, intimidation, and informal social control shape everyday behaviour, constrain disclosure, and normalise exposure to harm (McAlister et al., 2013). These dynamics are further reflected in children’s rights and inspection evidence, which consistently points to under-reporting, reduced visibility of exploitation, and safety concerns that limit both help-seeking and statutory engagement in some localities (NICCY, 2021; Criminal Justice Inspection Northern Ireland, 2026). Independent oversight bodies similarly emphasise that ongoing paramilitary intimidation and coercive control continue to affect community confidence and system responsiveness, reinforcing the distinctiveness of the Northern Ireland context for prevention and intervention (Independent Reporting Commission, 2025).

These outer-setting barriers do not undermine the implementation architecture; rather, they illuminate the limits of what any cross-government programme can achieve without consistent political backing, stable funding cycles, and broader system reforms. The EPPOC case suggests that replicability requires jurisdictions to consider not only what the model is, but where it is deployed and whether external volatility can be buffered long enough to sustain implementation.

### 4.3. Inner-Setting

The inner setting includes the structural, political, and cultural contexts through which the implementation process proceeds (Damschroder et al., 2009). EPPOC benefited from unusually strong inner-setting structures: cross-government governance, a shared outcomes and benefits framework, and a coordinating Programme Team with specialist skills. These created the conditions for sustained collaboration, consistent planning, and a coherent whole-system response. However, inner-setting barriers were also evident. Partial system ownership meant that some departments and agencies integrated EPPOC principles deeply, while others maintained distance due to competing priorities, operational pressures, or capacity constraints.

The mismatch between EPPOC investment and core statutory infrastructure also shaped sustainability. Services that should sit within departmental baselines were instead dependent on ring-fenced funding, limiting mainstreaming. The EPPOC case, therefore, highlights that a strong implementation architecture can compensate for, but not fully overcome, structural gaps in system readiness.

### 4.4. Individual-Level

CFIR emphasises the role that individuals play in implementation (Damschroder et al., 2009). EPPOC’s success was strongly influenced by practitioner capability and identification with trauma-informed, prevention-led values. Workforce development, mentoring, and organisational development support enabled frontline practitioners to manage complex youth violence, coercive control, and exploitation safely and consistently. These supports contributed directly to acceptability, fidelity, and feasibility.

Yet capability varied significantly across localities. Replicability, therefore, depends on whether jurisdictions can cultivate practitioner capability at scale and maintain support in settings where contested legitimacy or fear may otherwise inhibit safe practice.

### 4.5. Process Mechanisms

Finally, CFIR highlights that successful change requires an active change process (Damschroder et al., 2009). One of the most distinctive insights from this case is that programme management methodologies (particularly MSP) served as de facto implementation mechanisms. Planning structures, coordinated execution, benefits realisation, and iterative learning created a disciplined change process that resembles implementation science process guidance. These mechanisms strengthened adoption, feasibility, and fidelity, and sustained cross-agency collaboration even during periods of significant political instability.

The implication for replication suggests that jurisdictions do not necessarily need sophisticated implementation-science infrastructure to build an effective system architecture. Programme management tools, if applied with an implementation mindset, can generate many of the same mechanisms.

### 4.6. Transferability

EPPOC’s approach may hold lessons for other settings, particularly those facing similar complex, multi-faceted violence issues. The key elements that might be transferable include:

- a stable or protected authorising environment capable of buffering volatility;
- cross-government ownership rather than departmental containment to prevent siloed delivery and ensure shared accountability;
- a shared outcomes and benefits framework that aligns diverse interventions to system-level goals;
- the ability to build practitioner capability and maintain safe practice in coercive or contested contexts;
- a learning system that supports iteration, contextual adaptation and cross-agency sensemaking; and
- mechanisms for mainstreaming to reduce dependence on ring-fenced funding.

These conditions do not guarantee population-level impact, particularly in contexts characterised by coercion, structural disadvantage, or contested legitimacy, but they should provide other jurisdictions and initiatives with the tools to design structures with implementation success in mind, accounting for their local context.

### 4.7. Limitations

Although our analysis highlights several positive implementation outcomes, these should be interpreted cautiously. This research was looking narrowly at implementation strategies and outcomes, and we can say that the processes put in place functioned in ways that stakeholders believe are beneficial and which supported change. However, the study is based on secondary analysis of EPPOC documentation, evaluations, action research, and contribution analysis, interpreted by practitioner-analysts with deep positional knowledge. Although structured frameworks were used to mitigate bias, the analysis is shaped by the evidence available and the authors’ professional roles. The absence of experimental designs, uneven evaluation coverage across projects, and the complexity of attributing system-level change in volatile environments limit the ability to generalise findings. Nonetheless, the analysis offers real-world insight into system-level implementation in complex social systems.

Future research should consider comparative case studies or even experimental designs to test the impact of a strong implementation architecture on outcomes. Additionally, comparative research in other contexts could examine if the strategies identified here (like trauma-informed training and multi-agency governance) yield similar implementation benefits.

For practitioners and policymakers, the EPPOC case underscores the importance of investing (in the widest sense of the term) in the often ‘invisible’ aspects of reform (structures, relationships, shared learning) and not just in frontline activities.

## 5. Conclusions

This case study suggests that the programme’s implementation architecture—the structures and processes coordinating action—may represent a potentially transferable unit of innovation for other contexts tackling serious youth violence. EPPOC demonstrates that implementation architecture, rather than discrete interventions, may be the most portable unit of innovation for jurisdictions grappling with serious youth violence in complex environments. The Programme’s ability to maintain cross-government coordination, trauma-informed practice, and adaptive learning under conditions of coercion and volatility underscores the value of structured programme management as an implementation mechanism. The model’s replicability rests less on context-specific content and more on the disciplined, values-driven architecture that enabled the system to function over nearly a decade. While EPPOC illustrates what is possible, it also demonstrates the limits of programme-led system reform in the absence of stable political and fiscal conditions.

When assessed through implementation outcomes, EPPOC’s distinctive strengths are in acceptability and adoption in a context where labels linked to armed groups and/or conflict can undermine the legitimacy and engagement of youth violence interventions. The Programme’s deliberate framing shifts, which focused on harms rather than identities, adapted contested language, and positioned trauma-informed prevention as the shared value base, functioned as implementation actions that improved perceived fit across sectors and communities.

This case study is only a start in describing how policy recommendations from organisations like the World Health Organisation to take whole system approaches can be designed and implemented. This study has limitations insofar as it is a study of one case in a specific context. There is a need to build from this case study to find comparative examples to identify common and generalisable mechanisms to tackle youth violence. We have used implementation science as a comparative lens to provide an empirically and theoretically informed language to describe how EPPOC was implemented and how it achieved its implementation outcomes. However, whole-system approaches to youth violence are complex and context-dependent, and we need significantly more research on how they work in practice and how they navigate their own particular contextual challenges. The Northern Ireland case provides a hopeful example of how such an approach can be implemented, and highlights ingredients that may help others attempting similar reforms. Ultimately, turning the tide on serious youth violence will require both effective implementation architectures and rigorous evaluation of their impact.

## Data Availability

The data analysed in this study consist of publicly available programme documents, independent evaluations, oversight reports, and published population-level administrative statistics. No new datasets were generated for this study. Further information about the sources used is available in the References section.

## Appendix A. Implementation Strategies Used by EPPOC

**Table A1:** Utilise financial strategies.

Implementation Strategy	Used by EPPOC?	Brief Description of EPPOC’s Utilisation of Strategy
Fund and contract for the clinical innovation	Yes	EPPOC allocated and managed funding for multi-agency projects delivering trauma informed and preventative activity.
Access new funding	Yes	EPPOC secured new government funding through the Fresh Start Agreement and subsequent programme approvals.
Place innovation on fee for service lists/formularies	No	EPPOC did not use reimbursement based mechanisms.
Alter incentive/allowance structures	No	EPPOC did not alter financial incentives for practitioners or organisations.
Make billing easier	No	EPPOC did not make changes to billing processes.
Alter patient/consumer fees	No	EPPOC did not modify consumer fee structures.
Use other payment schemes	No	EPPOC did not use alternative payment models.
Develop disincentives	No	EPPOC did not develop financial disincentives for non-adoption.
Use capitated payments	No	EPPOC did not introduce capitated payment systems.

**Table A2:** Develop stakeholder interrelationships.

Implementation Strategy	Used by EPPOC?	Brief Description of EPPOC’s Utilisation of Strategy
Identify and prepare champions	Yes	EPPOC intentionally identified key individuals across departments and delivery organisations and supported them to promote the programme’s aims and drive cultural and operational change.
Organise clinician implementation team meetings	No	EPPOC did not centrally organise formal clinician implementation teams, although some delivery partners and key leads used similar mechanisms within their own projects. However, at a system level, we used this strategy with key delivery leads and leadership figures.
Recruit, designate, and train for leadership	Yes	EPPOC recruited and equipped departmental and project leaders to support shared ownership of outcomes and coordinate change across sectors.
Inform local opinion leaders	Yes	EPPOC engaged influential practitioners and community figures to reinforce understanding of programme priorities and encourage wider adoption.
Build a coalition	Yes	EPPOC built a cross departmental and cross sector coalition that mobilised collective resources, authority, and commitment to address paramilitary harm.
Obtain formal commitments	Yes	EPPOC secured formal departmental commitments to shared delivery responsibilities, governance structures, and agreed outcomes.
Identify early adopters	Yes	EPPOC identified delivery leads and organisations willing to adopt new approaches early, using their experience to shape broader implementation.
Conduct local consensus discussions	Yes	EPPOC facilitated cross agency and community discussions to build shared understanding of harms and agreement on appropriate interventions.
Capture and share local knowledge	Yes	EPPOC gathered practice insight from delivery partners and community organisations and shared this learning across the system.
Use advisory boards and workgroups	Yes	EPPOC used advisory groups, workstreams, and governance boards to gather multi stakeholder input and support decision making.
Use an implementation advisor	No	EPPOC did not use an external implementation advisor because its approach evolved from internal programme management rather than implementation science.
Model and simulate change	Yes	EPPOC used modelling and scenario testing to assess proposed changes and understand system wide impacts before introducing new activity.
Visit other sites	Yes	EPPOC learned from visits to other jurisdictions and programmes to understand comparable approaches to violence reduction and system reform.
Involve executive boards	Yes	EPPOC engaged executive boards and senior governance structures across departments to maintain oversight and ensure accountability.
Develop an implementation glossary	Yes	EPPOC developed shared terminology to support consistent understanding of trauma informed principles, outcomes, and programme mechanisms.
Develop academic partnerships	Yes	EPPOC partnered with academic institutions to draw in research expertise and strengthen evaluation and learning.
Promote network weaving	Yes	EPPOC strengthened cross sector relationships by intentionally connecting partners to support information sharing and collaborative problem solving.

**Table A3:** Change Infrastructure.

Implementation Strategy	Used by EPPOC?	Brief Description of EPPOC’s Utilisation of Strategy
Mandate change	Yes	EPPOC secured ministerial and departmental mandates that positioned the programme as a priority requiring cross government action.
Change record systems	Yes	EPPOC supported changes to data systems to improve shared visibility of harms, interventions, and outcomes.
Change physical structure and equipment	Yes	EPPOC enabled changes to physical spaces and equipment where required to support delivery partners and improve service access.
Create or change credentialing and/or licensure standards	No	EPPOC did not alter credentialing or professional licensing arrangements because these lay beyond programme remit.
Change service sites	Yes	EPPOC expanded or adjusted service locations to increase access for communities affected by paramilitary harm.
Change accreditation or membership requirements	No	EPPOC did not seek changes in accreditation or membership requirements for organisations.
Start a dissemination organisation	No	EPPOC did not create a dedicated dissemination body, relying instead on existing structures and cross government governance.
Change liability laws	No	EPPOC did not pursue legal liability reforms because these fell outside its operational scope.

**Table A4:** Provide Interactive Assistance.

Implementation Strategy	Used by EPPOC?	Brief Description of EPPOC’s Utilisation of Strategy
Facilitation	Yes	EPPOC provided facilitation through its central team, enabling problem solving, coordination, and support across departments and delivery partners.
Provide local technical assistance	No	EPPOC did not establish a local technical assistance model because projects were too varied for a centralised technical team to support.
Provide clinical supervision	Yes	Some EPPOC funded interventions included clinical supervision structures to support trauma informed practice.
Centralise technical assistance	No	EPPOC did not create a centralised technical assistance function due to the diversity of interventions and system level focus.

**Table A5:** Train and Educate Stakeholders.

Implementation Strategy	Used by EPPOC?	Brief Description of EPPOC’s Utilisation of Strategy
Conduct ongoing training	Yes	EPPOC funded extensive trauma informed training across statutory, voluntary, and community organisations.
Provide ongoing consultation	Yes	EPPOC provided sustained consultation through learning forums, governance structures, and cross departmental engagement.
Develop educational materials	Yes	EPPOC supported the development of trauma informed toolkits, e learning modules, guidance, and organisational development materials.
Make training dynamic	Yes	EPPOC delivered interactive, context sensitive, and adaptive training formats to meet varied organisational needs.
Distribute educational materials	Yes	EPPOC distributed training resources and materials across sectors.
Use train-the-trainer strategies	Yes	EPPOC invested in train the trainer models to build internal capacity and scale trauma informed practice.
Conduct educational meetings	Yes	EPPOC organised cross agency training sessions, briefings, and workshops to support understanding and adoption.
Conduct educational outreach visits	No	EPPOC did not use structured outreach visits as an implementation method.
Create a learning collaborative	Yes	EPPOC established learning forums and cross sector spaces for shared reflection and improvement.
Shadow other experts	Yes	EPPOC facilitated opportunities for practitioners and leaders to observe expertise in trauma informed and collaborative approaches.
Work with educational institutions	Yes	EPPOC collaborated with universities to strengthen training, research, and evaluation.

**Table A6:** Use evaluative and iterative strategies.

Implementation Strategy	Used by EPPOC?	Brief Description of EPPOC’s Utilisation of Strategy
Assess for readiness and identify barriers and facilitators	Yes	EPPOC assessed contextual barriers including trauma, coercive control, siloed systems, and resource constraints.
Audit and provide feedback	No	Some audit and feedback processes existed within individual projects, but EPPOC did not operate a comprehensive system level audit mechanism.
Purposefully reexamine the implementation	Yes	EPPOC periodically reconsidered its implementation model, especially during transitions between programme phases.
Develop and implement tools for quality monitoring	No	EPPOC did not create formal quality monitoring tools at system level, though some partners did so within their own organisations.
Develop and organise quality monitoring systems	No	EPPOC did not develop full system wide quality monitoring systems but supported more limited performance dashboards and reporting.
Develop a formal implementation blueprint	Yes	EPPOC developed a programme blueprint through MSP methodology, outlining governance, outcomes, and delivery structures.
Conduct local need assessment	Yes	EPPOC commissioned and used local needs assessments to inform portfolio design and prioritisation.
Stage implementation scale up	Yes	EPPOC adopted a phased approach, starting with early delivery mechanisms and later scaling to system level reform.
Obtain and use patients/consumers and family feedback	Yes	EPPOC used community and service user feedback within project level evaluation and in shaping portfolio learning.
Conduct cyclical small tests of change	Yes	EPPOC supported iterative piloting and refinement through portfolio projects, using test and learn cycles.

**Table A7:** Adapt and tailor to context.

Implementation Strategy	Used by EPPOC?	Brief Description of EPPOC’s Utilisation of Strategy
Tailor strategies	Yes	EPPOC tailored strategies across departments and localities based on differing needs, risks, and delivery conditions.
Promote adaptability	Yes	EPPOC promoted adaptability by embedding feedback loops and allowing projects to evolve as insights emerged.
Use data experts	Yes	EPPOC used data expertise from departmental analysts and cross Executive platforms to support monitoring and decision making.
Use data warehousing techniques	No—but attempts made	EPPOC attempted to develop shared data architecture but did not fully implement warehousing solutions due to technical and organisational barriers.

**Table A8:** Engage consumers.

Implementation Strategy	Used by EPPOC?	Brief Description of EPPOC’s Utilisation of Strategy
Involve patients/consumers and family members	Yes	EPPOC involved service users and communities through participatory evaluation, co design, and community feedback loops.
Intervene with patients/consumers to enhance uptake and adherence	No	EPPOC did not run clinical adherence interventions because its focus is system level rather than clinical pathways.
Prepare patients/consumers to be active participants	No	EPPOC did not deliver structured programmes designed to activate service users in clinical decision making.
Increase demand	No	EPPOC did not focus on increasing consumer demand for specific interventions.
Use mass media	Yes	EPPOC used communications and public engagement campaigns to raise awareness of harms and available support.

**Table A9:** Support clinicians.

Implementation Strategy	Used by EPPOC?	Brief Description of EPPOC’s Utilisation of Strategy
Facilitate relay of clinical data to providers	Yes	EPPOC improved data flows for some projects by supporting shared dashboards and cross agency information exchange.
Remind clinicians	No	EPPOC did not use reminder systems for practitioners.
Develop resource sharing agreements	Yes	EPPOC supported resource sharing arrangements across departments and partners.
Revise professional roles	No	EPPOC did not revise professional roles at system level.
Create new clinical teams	Yes	Some EPPOC funded interventions established new multidisciplinary teams to support trauma responsive practice.
